# Supplementary material for: Routing of the RAB6 secretory pathway towards the lysosome related organelle of melanocytes
Source: Nat Commun. 2017 Jun 13;8:15835. doi: 10.1038/ncomms15835 (PMC5474736; doi:10.1038/ncomms15835)
Supplement: Supplementary Information — Supplementary Figures and Supplementary Table [file ncomms15835-s1.pdf]

Supplementary Figure 1

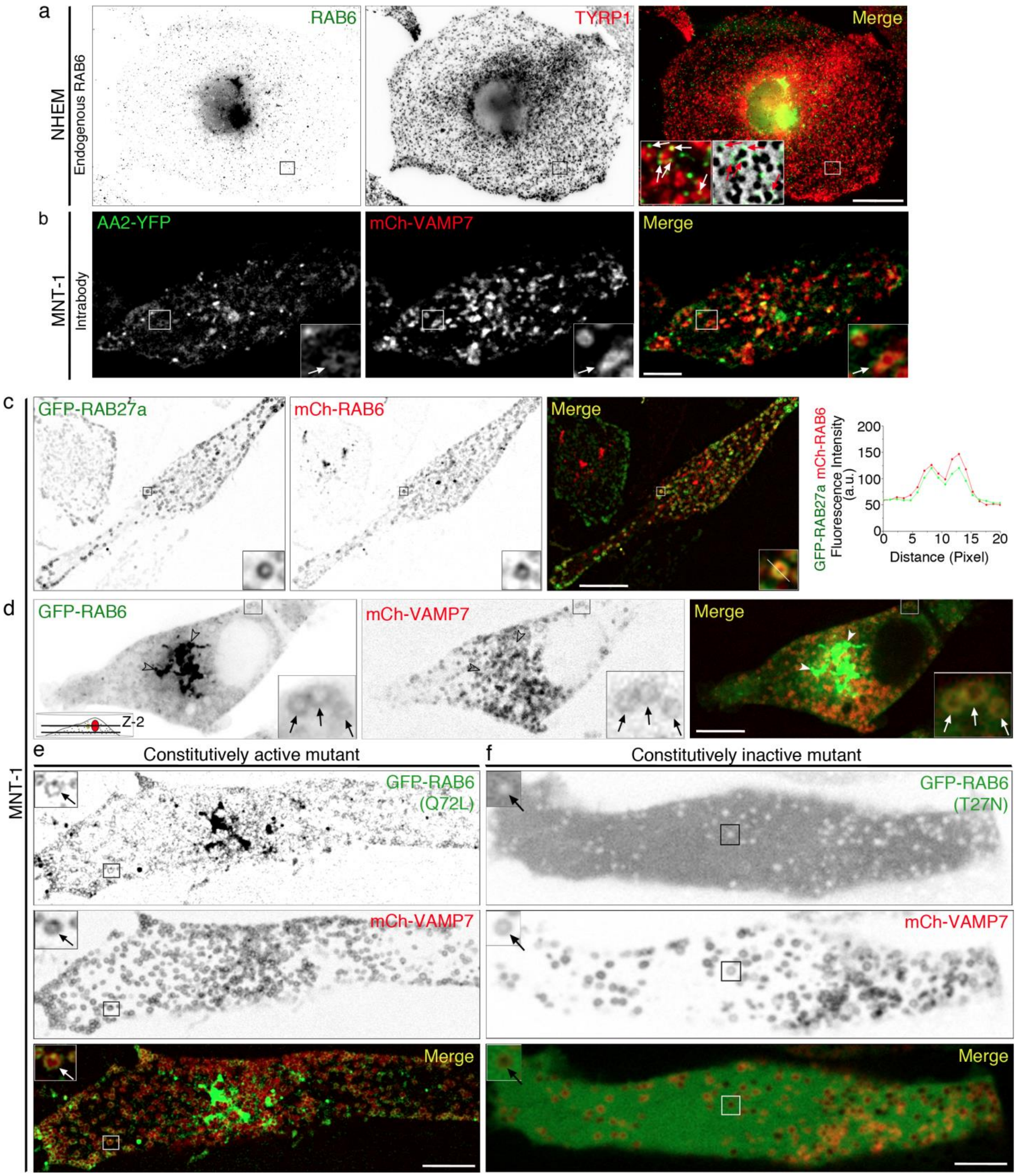

Supplementary Figure 1: RAB6 associates to melanosomes in a GTP-dependent manner

(**a**) IFM on Normal Human Epidermal Melanocytes (NHEM) stained for endogenous RAB6 and TYRP1 showing RAB6 association with TYRP1- and melanin-positive melanosomes (white and red arrows, respectively, 5X area). (**b**) Live imaging frame of MNT-1 cells co-expressing AA2-YFP (RAB6:GTP intrabody) and mCh-VAMP7. AA2-YFP localized as punctate or donut-like structure (arrow) co-distributed with mCh-VAMP7 (3X area, see also **Supplementary Movie 1**). (**c**) Live imaging frame of MNT-1 cells expressing GFP-RAB27a and mCh-RAB6. Corresponding intensity profile of RAB6 across melanosome was measured along the white line (5X area). (**d**) Live imaging frame of MNT-1 cells co-expressing GFP-RAB6 and mCh-VAMP7 (imaging plane Z-2 of the same cell as in Figure 1d) showing RAB6 distribution to the Golgi area (arrowheads) and to VAMP7-positive melanosomes (arrows, 5X area). (**e-f**) Live imaging frame of MNT-1 cells expressing GFP-RAB6 mutants and mCh-VAMP7 that showed melanosome-associated activated GFP-RAB6 (Q72L mutant, arrows, e), or cytosolic inactive mutant (T72N, f). Scale bars: 10 µm (a-f).

Supplementary Figure 2

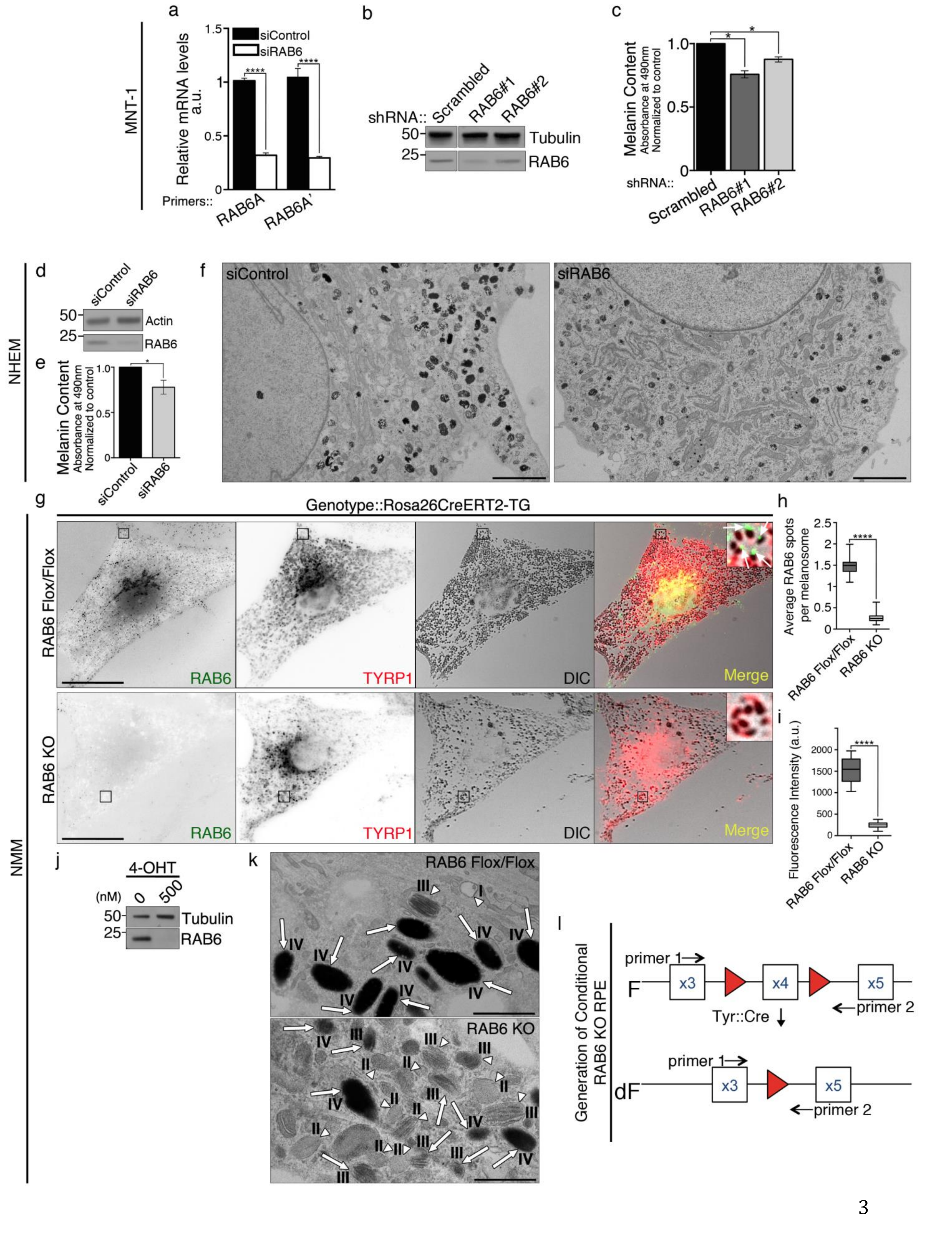

Supplementary Figure 2: *In vitro* or *in vivo* RAB6 extinction impacts pigmentation

(**a**) Relative mRNAs expression levels of RAB6 isoforms (A & A') by real time PCR of RAB6 siRNAs treated MNT-1 cells. Data are normalized to 26s rRNA as control and presented as mean ± SEM (3 independent experiments). (**b**) WB of control (scramble) or RAB6 shRNAs expressing MNT-1 stable cell lines; lysates were probed for RAB6 or Tubulin. (**c**) Melanin content estimation of cells treated as in b. Data are normalized to control and presented as means ± SEM (3 independent experiments). (**d**) WB of control- or RAB6-siRNA depleted NHEM probed for RAB6 or Actin. (**e**) Melanin content estimation of siRNA-treated NHEM as in (d). Data are normalized to control and presented as means ± SEM (3 independent experiments). (**f**) Representative electron micrograph by conventional EM on control- or RAB6-depleted NHEM cells. (**g**) IFM on NMM isolated from Rosa26CreERT2-TG mice treated (bottom, RAB6 KO) or not (top, RAB6 Flox/Flox) with 4-OHT (4-hydroxy tamoxifen) and stained for endogenous RAB6 and TYRP1. RAB6 localized in the vicinity of TYRP1- (red) and melanin-positive melanosomes (arrows), which disappeared upon 4-OHT treatment (5X area). (**h-i**) Quantification of the number of endogenous RAB6-positive structures associated to melanosomes (h) and overall RAB6 fluorescent intensity (i) in NMM treated as in (g) (90-100 melanosomes per cell, n=31-35 cells, 7 independent mice). (**j**) WB of ethanol- (untreated) or 4-OHT-treated NMM (as in (g)), probed for RAB6 or Tubulin as control. No change in RAB6 protein expression level was observed in *Rosa26CreERT2-TG*$^{-/-}$; *Rab6*$^{F/F}$ mouse melanocytes (data not shown). (**k**) High magnification EM image from cells as in Figure 2e showing immature melanosomes (arrowheads) and pigmented melanosomes (arrows). Note the increase of immature organelles in RAB6 KO NMM. (**l**) Diagram (not to the scale) of the floxed (F) and defloxed (dF) Rab6 locus. LoxP sequences (red triangle) flank Rab6 exon 4 (x4) of the floxed allele. After appropriate crosses with Tyr::Cre transgenic mice, Rab6 exon 4 is deleted. The mice genomic DNA was amplified using primers 1 and 2 (arrow 1 and 2). The exons are represented as rectangles and the black line corresponds to the intronic sequence. Scale bars: 5 µm (f), 20 µm (g), 500 nm (k); Two-tailed, unpaired t tests (a, c, e, h, j); *, $P < 0.05$; **** $P < 0.0001$; M.W. in kDa.

Supplementary Figure 3

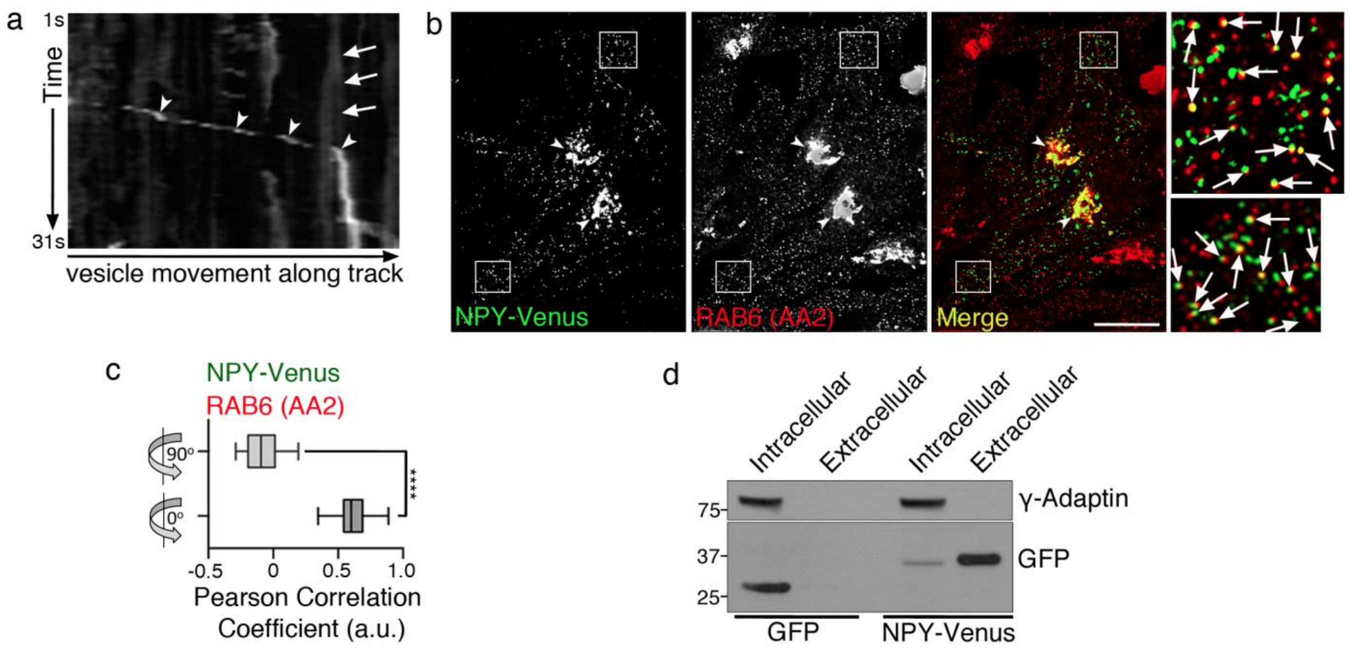

Supplementary Figure 3: Exogenously-expressed NPY co-distribute with RAB6 in MNT-1 cells

(**a**) Representative kymograph (track from **Supplementary Movie 4**) of a motile GFP-RAB6-positive vesicle (arrowheads), immobilizing in the vicinity of a melanosome (arrows). (**b**) NPY-Venus expressing MNT-1 cells stained for endogenous RAB6:GTP (AA2) showed co-distribution on Golgi (arrowheads) and peripheral vesicles (arrows, 5X area). (**c**) Pearson's correlation coefficient of NPY-Venus and RAB6:GTP. Correlation was lost when one of the channels was rotated by 90° (n=34 cells, 4 independent experiments). (**d**) WB of GFP or NPY-Venus expressing MNT-1 cell lysates and supernatant recovered 24h post-transfection and probed for GFP or γ-adaptin (subunit of adaptor protein complex 1, as intracellular protein control). Scale bar: 10 μm; Two-tailed, unpaired t tests; **** $P < 0.0001$; M.W. in kDa.

Supplementary Figure 4

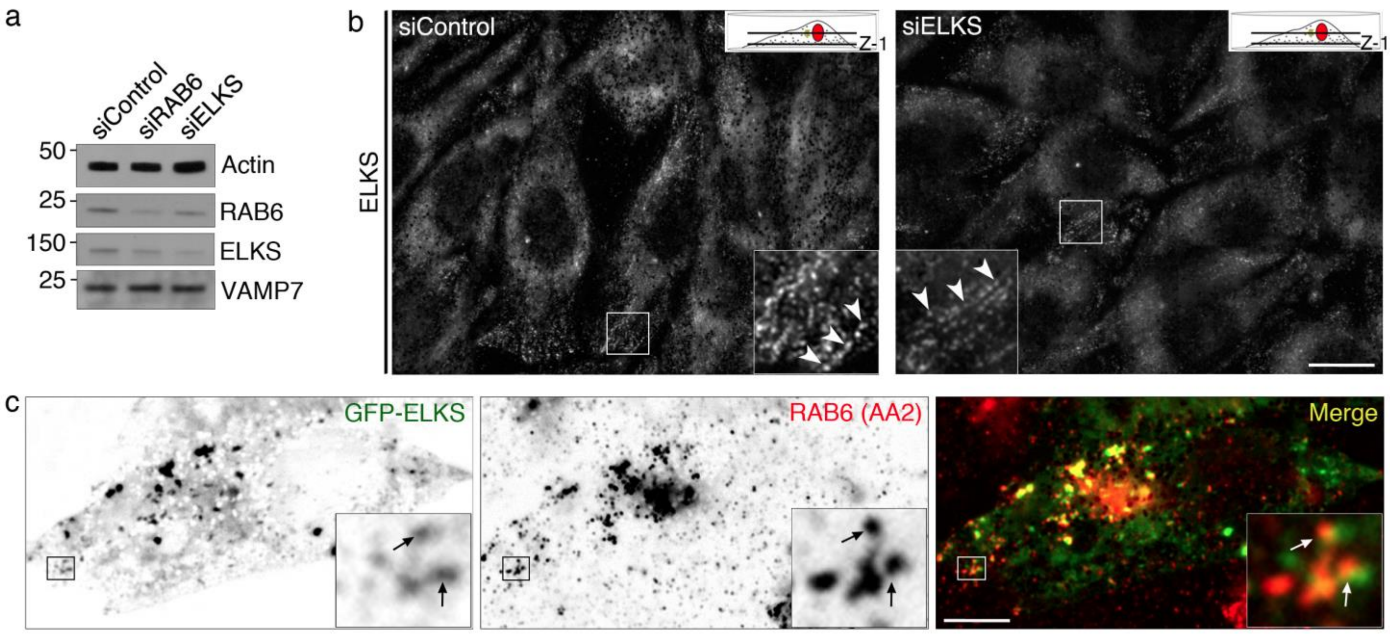

Supplementary Figure 4: ELKS associates with activated endogenous RAB6-positive vesicles and to structures close to plasma membrane.

(**a**) WB of MNT-1 cell lysates treated with indicated siRNAs and probed for RAB6, ELKS, VAMP7 and Actin. (**b**) IFM on control- or ELKS-depleted MNT-1 cells imaged close to the glass (basolateral imaging plane Z-1) showed ELKS-positive structures close to plasma membrane (arrowheads, 5X area). (**c**) IFM on GFP-ELKS expressing MNT-1 cells stained for endogenous activated RAB6 (AA2) showed co-distribution on peripheral structures (arrows, 5X area). Scale bar: 20 µm (b, c); M.W. in kDa.

Supplementary Figure 5

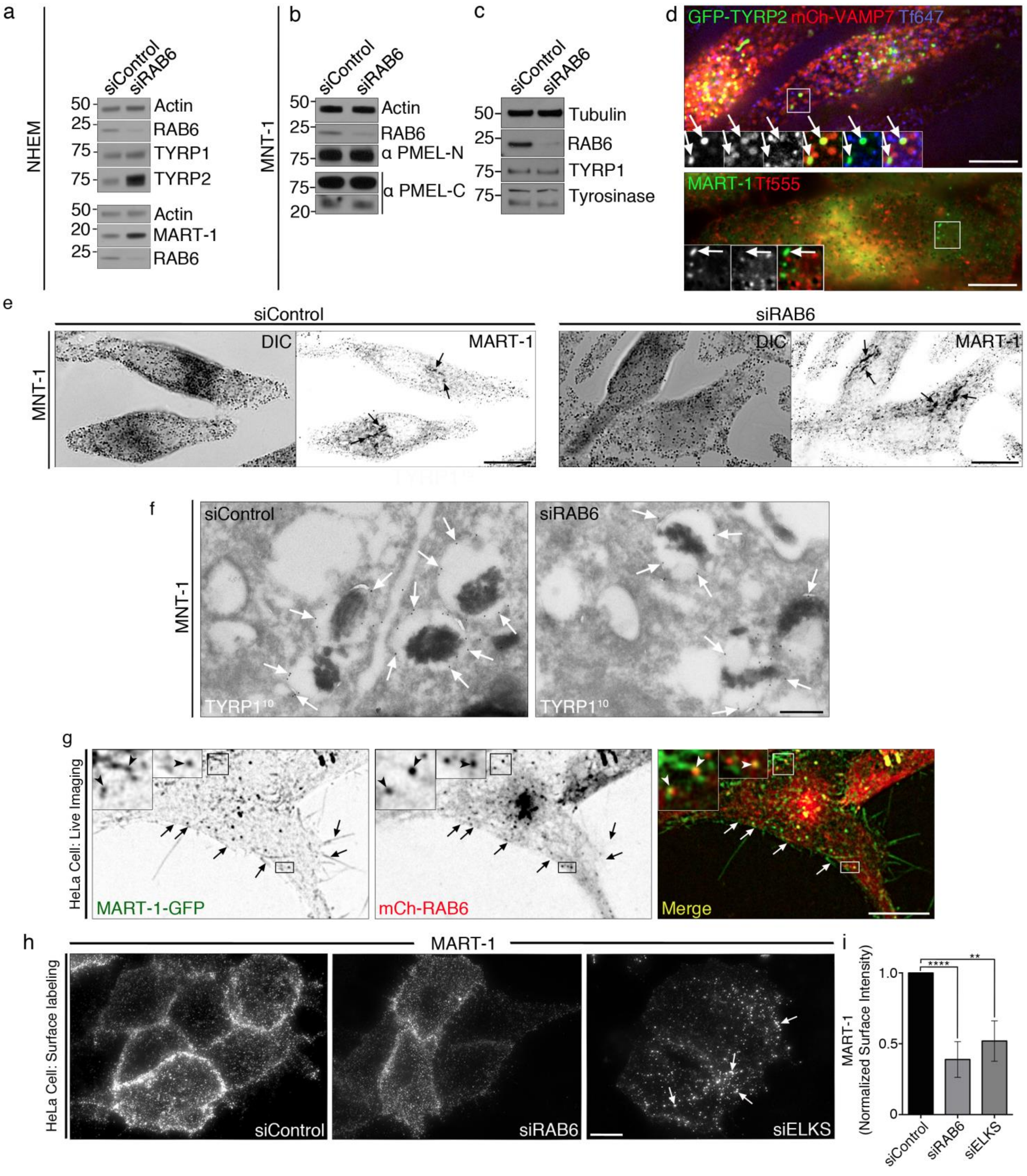

Supplementary Figure 5: MART-1 and TYRP2 are specific cargoes of the RAB6/ ELKS-dependent route.

(**a**) WB of control- or RAB6-depleted NHEM lysates probed for respective antibodies. (**b-c**) WB of control- or RAB6-depleted MNT-1 cell lysates showing no alterations in PMEL processing (b) and/ or TYRP1, Tyrosinase expression levels (c). (**d, top**) Live imaging frame of GFP-TYRP2 and mCh-VAMP7 co-expressing Tf-A647-internalized MNT-1 cells showing the co-distribution of TYRP2 with melanosomes (arrows, 2X area), but not with Tf-positive endosomes. (**Bottom**) IFM on TfA555-internalized MNT-1 cells stained for endogenous MART-1 did not show co-localization (arrows, 2X area). (**e**) IFM on control-or RAB6-depleted MNT-1 cells stained for endogenous MART-1 that accumulated at the Golgi area (arrows) upon RAB6 depletion, quantified in Figure 6h. (**f**) Immuno-labeling of ultrathin cryosection of control- or RAB6-depleted MNT-1 cells using TYRP1 antibodies (PAG-10 nm). TYRP1 localized to the limiting membrane of melanosomes (arrows). (**g**) Live imaging frame of HeLa cells co-expressing MART-1-GFP and mCh-RAB6. MART-1-GFP associated to the cell edges (arrows) and co-distributed with RAB6 on peripheral vesicles (arrowheads, 2X area). (**h**) MART-1 surface staining (using MART-1 N-terminal extracellular domain specific antibody) on non-permeabilised control-, RAB6- or ELKS-depleted HeLa cells expressing MART-1 untagged construct. RAB6 depletion decreased the overall staining, while the distribution appeared scattered (arrows) in ELKS depleted cells. (**i**) Quantification of cell surface staining of MART-1 in cells treated as in (h). Data are normalized to control and presented as mean ± SEM (n=55-78 cells, 2 independent experiments). Scale bars: 10 μm (d, e, g, h), (f) 200 nm; Two tailed, Unpaired t test (i); **, $P < 0.01$; ****, $P < 0.0001$; M.W. in kDa.

Supplementary Figure 6: Uncropped scans of the most important blots.

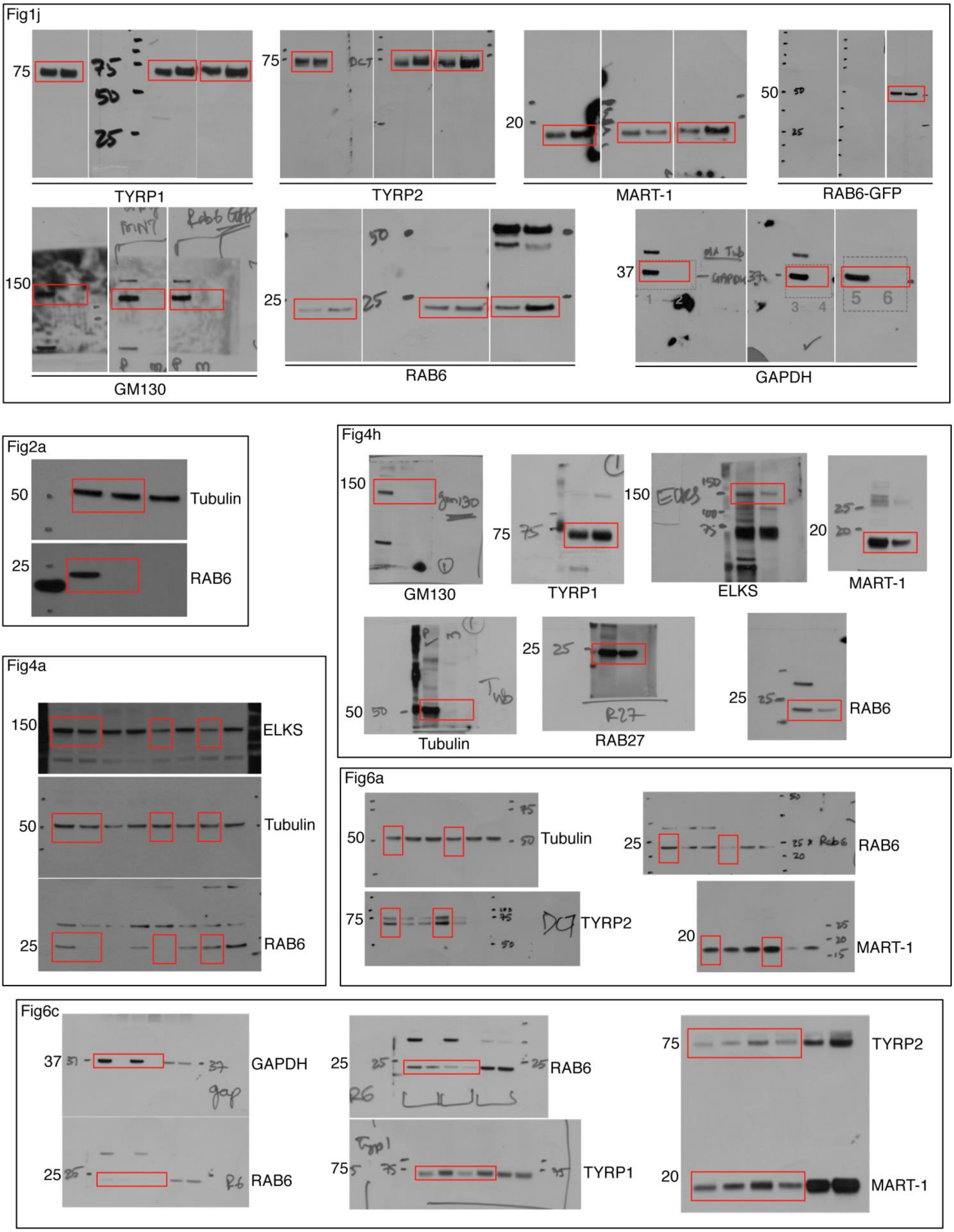

Supplementary Table 1: Analysis of Rab6 vesicles dynamics (mean ± SEM)

siRNA	Cell	Vesicle	Track Time (sec)	Track time in Pause (%)	Average Velocity (µm/sec)	Pausing Vesicle (vesicle)	Pausing vesicle at melanosome (% of total pause)
**-**	12	80	15 ± 0.70	31 ± 2.6	0.88 ± 0.04	84% (69)	-
**Control**	16	112	15 ± 0.58	34 ± 2.2	0.84 ± 0.04	87.5% (98)	70.8%
**ELKS**	16	88	16 ± 0.74	18 ± 2.0	0.86 ± 0.05	63% (56)	37.7%
